# Molecular Machines Determining the Fate of Endocytosed Synaptic Vesicles in Nerve Terminals

**DOI:** 10.3389/fnsyn.2016.00010

**Published:** 2016-05-12

**Authors:** Anna Fassio, Manuela Fadda, Fabio Benfenati

**Affiliations:** ^1^Department of Experimental Medicine, University of GenoaGenoa, Italy; ^2^Center of Synaptic Neuroscience and Technology, Istituto Italiano di TecnologiaGenova, Italy

**Keywords:** presynapse, synaptic vesicle, cdk5, calcineurin, recycling, small GTPases, Rab, Arf

## Abstract

The cycle of a synaptic vesicle (SV) within the nerve terminal is a step-by-step journey with the final goal of ensuring the proper synaptic strength under changing environmental conditions. The SV cycle is a precisely regulated membrane traffic event in cells and, because of this, a plethora of membrane-bound and cytosolic proteins are devoted to assist SVs in each step of the journey. The cycling fate of endocytosed SVs determines both the availability for subsequent rounds of release and the lifetime of SVs in the terminal and is therefore crucial for synaptic function and plasticity. Molecular players that determine the destiny of SVs in nerve terminals after a round of exo-endocytosis are largely unknown. Here we review the functional role in SV fate of phosphorylation/dephosphorylation of SV proteins and of small GTPases acting on membrane trafficking at the synapse, as they are emerging as key molecules in determining the recycling route of SVs within the nerve terminal. In particular, we focus on: (i) the cyclin-dependent kinase-5 (cdk5) and calcineurin (CN) control of the recycling pool of SVs; (ii) the role of small GTPases of the Rab and ADP-ribosylation factor (Arf) families in defining the route followed by SV in their nerve terminal cycle. These regulatory proteins together with their synaptic regulators and effectors, are molecular nanomachines mediating homeostatic responses in synaptic plasticity and potential targets of drugs modulating the efficiency of synaptic transmission.

## Introduction

The synaptic vesicle (SV) cycle is the most highly regulated membrane traffic event in cells and the proteins involved, as well as the arrays of protein-protein interactions that guarantee the fidelity of the process, are getting increasingly clear.

Kinases and phosphatases, as well as small GTPases and their effectors, are emerging as molecular machines acting at the synapse to regulate synaptic function and plasticity by rapidly modulating several synaptic targets and adapting the synapse to the needs of the network.

The high tunability of synaptic strength is obtained presynaptically from changes in quantal size, SV availability for release and number of active synapses, and all these parameters are directly or indirectly controlled by the cycling fate of SVs.

Here we provide an update on two molecular machines recently reported to act on the fate of recycling SVs in the nerve terminal: the cyclin-dependent kinase 5 (cdk5)/calcineurin (CN) system with its multiple synaptic substrates and the small GTPase Rab and ADP ribosylation factor (Arf) systems with their relative regulators and effectors.

## The cdk5/CN System

Kinases and phosphatases post-translationally regulate the function of a plethora of proteins and play a major role in regulating cellular functions. Phosphoproteins are highly expressed at the synaptic terminal together with dedicated kinases and phosphatases, and their activity regulates many aspects of SV cycling. The role of phosphoproteins in SV cycling has been extensively reviewed elsewhere (Valtorta and Benfenati, 1994). We here focus on one of the presynaptically expressed kinases, cdk5, and the cognate phosphatase, CN, as they act as key molecules in regulating the strength of neurotransmission by acting on SVs availability for release and endocytic recovery of SV at the plasma membrane after exocytosis.

The first evidence for a central role of the cdk5/CN system at the presynapse derives from the observation that several proteins involved in various steps of the SV cycle are specific substrates for cdk5/CN, namely munc18-1, septin5, Pictaire, synapsin, amphiphysin, dynamin and synaptojanin (reviewed in Su and Tsai, 2011). From a functional point of view, it is possible to distinguish between effects on exocytosis and endocytosis, keeping in mind that the two processes are intimately connected and both regulated by calcium waves in the terminal (Wu et al., 2014; Leitz and Kavalali, 2015). SV exocytosis is regulated by cdk5 phosphorylation of Munc18 that allows syntaxin1 to participate in the SNARE complex and SV fusion to occur (Shuang et al., 1998; Fletcher et al., 1999). A similar effect has been described for the phosphorylation of the cytoskeletal protein septin5 by cdk5, which decreases binding of septin5 to the SNARE protein syntaxin1 and regulates neurotransmitter release (Taniguchi et al., 2007; Amin et al., 2008). The cdk5 substrate Pictaire has also been proposed to regulate exocytosis by phosphorylating N-Ethylmaleimide-Sensitive Factor (NSF) and regulating the ability of NSF to oligomerize (Liu et al., 2006). Additional roles for cdk5/CN in the process of exocytosis came from the observation, made both *in vitro* and *in vivo*, that N-type calcium channels (Cav2.2) are substrates for the two enzymes (Su et al., 2012; Kim and Ryan, 2013). Although the impact of phosphorylation on the properties of the Cav2.2 channel is still controversial, it has been nicely demonstrated that the cdk5/CN balance regulates the final steps of exocytosis by potently controlling the action potential-driven calcium influx and therefore the probability of release (Kim and Ryan, 2013).

**Figure 1:**
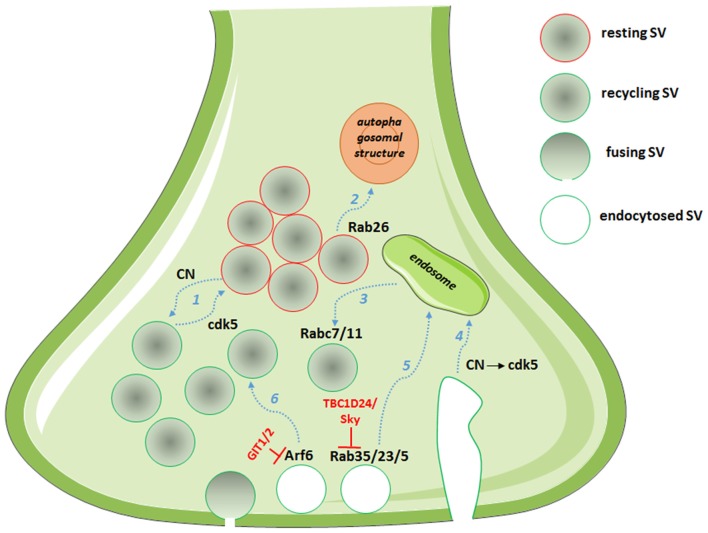
**Picture showing the multiple roles of cyclin-dependent kinase 5 (cdk5)/calcineurin (CN) and Rab/ADP-ribosylation factor (Arf) system in defining the route for endocytosed SVs.** Dashed arrows in light blue represent synaptic vesicle (SV) routes: (1) partitioning into pools; (2) degradation; (3) post-endosomal trafficking; (4) endosomal sorting after bulk endocytosis; (5) endosomal trafficking; and (6) direct recycling. In red, the GTPase activating proteins (GAPs) with the described functions in controlling the SV fate.

On the endocytosis side, an array of CN substrates involved in the SV retrieval process are known among the main molecular actors of SV cycling (Marks and McMahon, 1998). These proteins have been collectively named dephosphins, as their dephosphorylation is induced by calcium increase during stimulation (Cousin and Robinson, 2001). Both their dephosphorylation and subsequent phosphorylation by cdk5 is required for SV retrieval in central nerve terminals (Clayton et al., 2007). Different mechanisms of SV endocytosis operate at central synapses, namely fast endocytosis, clathrin-dependent endocytosis and bulk endocytosis (Kononenko and Haucke, 2015). However, how the various modes of endocytosis are interconnected and differently regulated is still a matter of investigation. The cdk5/CN activity on dephosphins has been reported to selectively regulate slow, activity-dependent bulk endocytosis, with no effect on fast endocytosis. The slow form of endocytosis predominantly occurs during sustained activity, requires traffic of SVs via the endosomal compartment and repopulates the recycling pool, which is only released during intense stimulation after the complete depletion of the readily releasable pool (Evans and Cousin, 2007; Cheung and Cousin, 2013). Interestingly, cdk5/CN activities concomitantly control which fraction of SVs partitions into the recycling or the release-reluctant resting pool (Kim and Ryan, 2010; Marra et al., 2012) and the SV protein Synapsin I has been identified as the main cdk5/CN substrate in mediating this effect (Verstegen et al., 2014). In particular, cdk5-phopshorylated Synapsin I sequesters recycling SVs in the release-reluctant resting pool by clustering SVs and increasing their association with actin filaments. The dual effect on bulk endocytosis and SV pool partitioning suggests that the system is involved in determining the fate of endocytosed SVs; in particular, it seems that during sustained activity bulk endocytosis proceeds via sequential CN/cdk5 activation, resulting in endosomal recycling of SVs and in the capture of the newly formed SVs into the release-reluctant pool (Figure 1). Moreover, the balance of cdk5/CN activities broadly varies between synapses, setting both the tone of N-type calcium channels and the ratio of recycling vs. reluctant SVs and resulting in synapse heterogeneity from silent to strongly active synapses (Kim and Ryan, 2010, 2013; Verstegen et al., 2014).

As a result of the multiple targets at the presynapse, the cdk5/CN nanomachine represents a master regulator of synaptic homeostasis and participates in the scaling of synaptic strength to compensate for the effects of sustained hypo- or hyperactivity (Seeburg et al., 2008; Kim and Ryan, 2010; Mitra et al., 2011; Peng et al., 2013). Chronic silencing of firing strongly downregulates nerve terminal cdk5 with the result of recruiting release reluctant SVs to the recycling pool. This process, which appears to be mediated by changes in the phosphorylation state of Synapsin I, can activate previously silent synapses and change the release potential of already active synapses (Kim and Ryan, 2010; Verstegen et al., 2014). Alterations of cdk5 activity are associated with a broad range of neurological disorders (see for reviews Cheung and Ip, 2012; McLinden et al., 2012) and in particular the cdk5-mediated homeostatic synaptic response has been recently involved in early Alzheimer’s like synaptic pathology (Sheng et al., 2015).

## Rab and Arf Systems

Rab and Arf family proteins are master regulators of membrane trafficking and are involved in all steps of vesicular transport. As all GTPases, these proteins function as molecular switches by cycling between active guanosine triphosphate (GTP)-bound and inactive guanosine diphosphate (GDP)-bound states. Their cycling is regulated by two families of regulatory proteins, namely guanine nucleotide exchange factors (GEFs) and GTPase activating proteins (GAPs). GEFs function as activators by facilitating the conversion from a GDP-bound form to a GTP bound form, whereas GAPs function as repressors by enhancing GTP hydrolysis. The vast number of Rab and Arf proteins and the multiple GAPs and GEFs for each isoform make the small GTPase family an ideal spatial and temporal regulator of virtually every aspect of membrane trafficking (Stenmark, 2009). As SV cycling is the prototype of an intensely regulated membrane traffic event, Rab and Arf proteins, together with their regulators and effectors, are emerging as molecular nanomachines regulating specific steps of the cycle.

More than 30 distinct Rabs were indeed identified by proteomic analysis in highly purified SV fractions (Takamori et al., 2006) and the three more abundant Rabs (Rab3, 7 and 5) were quantified in isolated terminals and found to represent 0.776, 0.135 and 0.025% of total synaptic protein, a percentage comparable with other fundamental protein for presynaptic physiology such as, for example, the calcium sensor synaptotagmin1 (Wilhelm et al., 2014). The role of the Rabs involved in exocytosis, such as the secretory Rabs Rab3a/3b/3c and Rab27b, and the role of some of the Rabs involved in SV recycling, such as Rab4, 5, 10, 11b and 14, has been reviewed and recently commented elsewhere (Sudhof, 2004; Pavlos et al., 2010; Pavlos and Jahn, 2011; Giorgini and Steinert, 2013; Rizzoli, 2014). Here we focus on additional small GTPases recently described to participate in SV cycling, particularly in the steps defining fate of SVs after recovery.

### Rab35

A role for Rab35 in SV cycling originated from the identification of TBC1D24, an epilepsy gene involved in neuronal development and protein partner of the small GTPase Arf6 (Corbett et al., 2010; Falace et al., 2010, 2014). Studying the *Drosophila* mutants for TBC1D24/Skywalker, Uytterhoeven et al. (2011) revealed a strong presynaptic phenotype with a larger readily releasable pool of SVs and a dramatic increase in basal neurotransmitter release at neuromuscular junction (NMJ) synapses. They reported TBC1D24 to act as a GAP for Rab35 and proposed that active GTP-bound Rab35 favors endosomal sorting of SV proteins and replacement of dysfunctional SV components. Rab5 and Rab23 were also described to exert similar effects on endosomal trafficking of SVs, although TBC1D24 seems to act as a selective GAP for Rab35. This is the first description of a synaptic machinery dedicated to the control of the quality of proteins in SV cycling (Figure 1, Uytterhoeven et al., 2011). Moreover, using a functional screen to assess the impact of a battery of constitutively active Rabs on SV cycling, the same authors identified Rab7 and Rab11 as putative regulators of post-endosomal trafficking of SVs (Figure 1, Uytterhoeven et al., 2011). Whether a similar mechanism also operates at mammalian central synapses remains to be investigated.

### Rab26

By investigating the molecular mechanisms leading to synapse elimination, Binotti et al. (2015) recently described a role for the small GTPase Rab26 in directing SVs into pre-autophagosomal structures, thus proposing a novel pathway for the degradation of SVs. The small GTPase was found in a subset of presynaptic terminals associated with marked clustering of SVs and is believed to promote SV clustering via a still unknown mechanism. Rab26, in its active GTP-bound state, was also reported to recruit Atg16L1 as an effector, representing a link between SV cycling and autophagy (Figure 1, Binotti et al., 2015).

### Arf

The Arf proteins are a family of six small, ubiquitously expressed GTP-binding proteins (Donaldson and Jackson, 2011) that can be divided into three classes, based on sequence identity. Class I Arf proteins (Arf1, Arf2 and Arf3) regulate the assembly of various types of “coat” complexes onto budding vesicles along the secretory pathway and activate lipid-modifying enzymes (Bonifacino, 2004); Class II Arf proteins (Arf4 and Arf5), whose function is still unclear; and Arf6, which is the sole member of class III Arf proteins known to regulate endosomal membrane traffic and structural organization at the cell surface (D’Souza-Schorey and Chavrier, 2006). Other proteins that structurally resemble Arf proteins are the Arf-like (Arl) proteins, the Ras-related protein-1 (SAR1p) and the Arf-related protein ARFRP1.

Although Arf proteins are less abundant in purified SVs or isolated nerve terminals as compared to other small GTPases, the Arf analogs Arl10b and Arl10c and the Arf-interacting protein arfaptin 2 have been identified as components of purified SVs (Takamori et al., 2006). Some of the Arf proteins have been described to play a role in the definition of SV fate at the synapse.

The class III Arf protein Arf6, involved in constitutive trafficking between the plasma membrane and early endosomes and actin dynamics, has been reported to increase basal synaptic transmission at the *Xenopus* NMJ (Ashery et al., 1999), to regulate the assembly of the clathrin-coat complex during SV endocytosis (Krauss et al., 2003) and to play a role in the SV recycling pathway (Tagliatti et al., 2016). Knockdown of Arf6 in rat hippocampal neurons results in a strong presynaptic phenotype, with decreased SV density, accumulation of endosomal structures in the terminal and increased functional releasable SVs docked to the plasma membrane. Arf6 appears to act as a molecular determinant in the formation of the readily releasable pool of SVs and in the sorting of endocytosed SVs to direct recycling, rather than through the endosomal compartment (Tagliatti et al., 2016, Figure 1). Interestingly, the observed Arf6-knockdown phenotype is reminiscent of the synaptic phenotype for the constitutively active Rab35 at the *Drosophila* NMJ (Uytterhoeven et al., 2011), suggestive of a functional interplay of the two small GTPases at the presynaptic compartment, as reported in different cellular systems (Allaire et al., 2013; Miyamoto et al., 2014). Although no precise synaptic role for Arf1 has been proposed, the protein in its active GTP-bound form has been described to regulate vesicle budding in PC12 cells (Faúndez et al., 1997), suggesting that also the prototype class I Arf may act in SV cycling pathway.

In support for a presynaptic role for Arf proteins, the Arf-like small G protein, Arl-8, has been identified as a critical regulator of presynaptic patterning and axonal transport in *C. elegans* (Klassen et al., 2010) and active GTP-bound Arl-8 were reported to act as an effector of the anterograde motor UNC-104/KIF1A (Wu et al., 2013). The Arl-8 GTP/GDP cycle is therefore proposed as a switch to control the association/dissociation of SV precursors from microtubule motor proteins, thus ensuring the proper delivery of novel presynaptic components at nerve terminals (Wu et al., 2013).

### GAPs and GEFs

Considering the prominent roles of Rab and Arf proteins at the synaptic terminal, it is noteworthy the lack of data on synaptic GAPs and GEFs that dynamically regulate their activity and potentially represent targets to finely modulate neurotransmission in both homeostatic and hebbian plasticity (Table 1). In addition to the above mentioned role for the Rab35-GAP TBC1D24 (Uytterhoeven et al., 2011), the specific GAP for Arf6, G-protein coupled receptor kinase 2 interacting protein (GIT) has been involved in the organization of the cytomatrix of the active zone (Kim et al., 2003) and recently reported to play multiple roles at the presynapse (Podufall et al., 2014; Montesinos et al., 2015). Podufall et al. (2014) analyzed the localization of GIT at hippocampal glutamatergic synapses and at the *Drosophila* NMJ by employing SD-dSTORM high-resolution microscopy and revealed that the protein localizes at the periphery of the active zone. Indeed, GIT interacts with the endocytic adaptor stoninB and regulates the localization and function of stoninB at the presynaptic site. A *Drosophila* GIT mutant showed accumulation of endosomal structures and vacuoles and marked defects in post-stimulus SV endocytosis and/or re-acidification (Podufall et al., 2014). Although confirming a clear presynaptic function for the GAP of Arf6, studies in mammalian synapses revealed different roles for GIT1 and the other mammalian isoform GIT2. GIT1 and GIT1/GIT2 knocked out calyx of Held synapses showed a markedly increased initial release probability that was not associated with changes in the size of the readily releasable pool or in voltage-dependent calcium channel activity. Although some discrepancies in the reported results exist, and the precise mechanisms of the synaptic actions of GIT are still to be clarified (Montesinos et al., 2015), the small GTPase Arf6 and the two Arf6 regulators, TBC1D24 and GIT, are emerging as synaptic nanomachines operating at the presynaptic site to define both release probability and SV recycling pathways.

**Table 1:** **Small GTPases playing a role at the presynaptic terminal**.

Small GTPases	Presynaptic role	Presynaptic GAP and GEF	Reference
Rab3a,b,c	Docking/priming	RAb3GAP, Rab3GEP…	Geppert et al. (1994, 1997); Yamaguchi et al. (2002), Giovedì et al. (2004a,b), Schlüter et al. (2004), Sakane et al. (2006), Pavlos et al. (2010), and Pavlos and Jahn (2011)
Rab4	SV recycling/sorting	To be determined	Pavlos and Jahn (2011)
Rab5a,b	SV recycling/sorting	To be determined	de Hoop et al. (1994), Fischer von Mollard et al. (1994), Shimizu et al. (2003), Wucherpfennig et al. (2003), Star et al. (2005), Hoopmann et al. (2010), Pavlos and Jahn (2011), and Uytterhoeven et al. (2011)
Rab7	SV recycling/sorting	To be determined	Pavlos and Jahn (2011) and Uytterhoeven et al. (2011)
Rab10	SV recycling/sorting	To be determined	Pavlos and Jahn (2011)
Rab11b	SV recycling/sorting	To be determined	Pavlos and Jahn (2011), Steinert et al. (2012), and Giorgini and Steinert (2013)
Rab14	SV recycling/sorting	To be determined	Pavlos and Jahn (2011)
Rab23	SV recycling/sorting	To be determined	Uytterhoeven et al. (2011)
Rab26	SV degradation	To be determined	Binotti et al. (2015)
Rab27b	Docking/priming	To be determined	Pavlos et al. (2010) and Pavlos and Jahn (2011)
Rab35	SV recycling/sorting	TBC1D24, Connecden1…	Allaire et al. (2006) and Uytterhoeven et al. (2011)
Arf1	SV budding	Arf1GAP…	Faúndez et al. (1997)
Arf6	SV recycling/sorting	GIT, Centaurin…	Ashery et al. (1999), Krauss et al. (2003), Homma et al. (2014), Podufall et al. (2014), Montesinos et al. (2015), and Tagliatti et al. (2016)
Arl8	SV component transport	To be determined	Klassen et al. (2010) and Wu et al. (2013)

The ArfGAP1, acting as a GAP for Arf1, has been found to interact and regulate the activity of Leucine-rich repeat kinase 2 (LRRK2, Xiong et al., 2012), whose gene is mutated in Parkinson’s disease patients, and known to play multiple roles in the presynaptic compartment (see for a review Belluzzi et al., 2012). LRRK2 kinase activity was also recently described to function as a Rab5b GAP, negatively regulating Rab5b signaling (Yun et al., 2015). In addition to LRRK2, huntingtin protein, dysfunctional in Huntigton’s disease, has been proposed to function as a Rab regulator. Indeed, huntingtin was found in a complex acting as a GEF for Rab11 (Li et al., 2008), and Rab11 overexpression rescued the synaptic dysfunction associated with both huntingtin and synuclein mutations in *Drosophila* (Steinert et al., 2012; Breda et al., 2015).

## Concluding Remarks

The cdk5/CN system is a master regulator of synaptic strength and synaptic functional heterogeneity by modulating calcium signaling and SV distribution in the nerve terminal. The system is highly regulated by homeostatic plasticity and represents a target for research on the molecular markers of synaptic dysfunctions and on the design of novel drugs (Shah and Lahiri, 2014; Sheng et al., 2015).

For the Rab/Arf system, we expect that additional GAP and GEF functions at the synapse will be clarified in the near future, together with the role of specific GTPase effectors in synaptic function. The synaptic targeting and/or the activity modulation of GAP and GEF will emerge as main factors in synaptic plasticity processes and in the pathogenesis of synaptic dysfunctions. Interestingly, several mutations in genes encoding proteins belonging to the Rab and Arf small GTPases families, or proteins regulating their GTP-binding cycle, have been recently described as causative for inherited neurological diseases (Falace et al., 2010; Shoubridge et al., 2010; Rauch et al., 2012; Seixas et al., 2013; D’Adamo et al., 2014; Fine et al., 2015; Kalscheuer et al., 2016), making the exploitation of their function at the synapse a prerequisite for the design of effective therapeutic strategies.

## Author Contributions

AF drafted the article and prepared the figure. MF assisted in drafting the article. FB drafted and revised the article.

## Conflict of Interest Statement

The authors declare that the research was conducted in the absence of any commercial or financial relationships that could be construed as a potential conflict of interest.
